# Phosphorylated Aβ peptides in human Down syndrome brain and different Alzheimer’s-like mouse models

**DOI:** 10.1186/s40478-020-00959-w

**Published:** 2020-07-29

**Authors:** Sathish Kumar, Cynthia A. Lemere, Jochen Walter

**Affiliations:** 1 Department of Neurology, University of Bonn, 53127 Bonn, Germany; 2 Ann Romney Center for Neurologic Diseases, Brigham and Women’s Hospital, Harvard Medical School, Boston, MA 02115 USA

**Keywords:** Alzheimer’s disease, Amyloid β peptide, Cerebral amyloid angiopathy, Down syndrome, Modified Aβ, Mouse models, Phosphorylation, Post-translational modification

## Abstract

The deposition of neurotoxic amyloid-β (Aβ) peptides in extracellular plaques in the brain parenchyma is one of the most prominent neuropathological features of Alzheimer’s disease (AD), and considered to be closely related to the pathogenesis of this disease. A number of recent studies demonstrate the heterogeneity in the composition of Aβ deposits in AD brains, due to the occurrence of elongated, truncated and post-translationally modified Aβ peptides that have peculiar characteristics in aggregation behavior and biostability. Importantly, the detection of modified Aβ species has been explored to characterize distinct stages of AD, with phosphorylated Aβ being present in the clinical phase of AD. People with Down syndrome (DS) develop AD pathology by 40 years of age likely due to the overproduction of Aβ caused by the additional copy of the gene encoding the amyloid precursor protein on chromosome 21. In the current study, we analysed the deposition of phosphorylated and non-phosphorylated Aβ species in human DS, AD, and control brains. In addition, deposition of these Aβ species was analysed in brains of a series of established transgenic AD mouse models using phosphorylation-state specific Aβ antibodies. Significant amounts of Aβ phosphorylated at serine residue 8 (pSer8Aβ) and unmodified Aβ were detected in the brains of DS and AD cases. The brains of different transgenic mouse models with either only human mutant amyloid precursor protein (APP), or combinations of human mutant APP, Presenilin (PS), and tau transgenes showed distinct age-dependent and spatiotemporal deposition of pSer8Aβ in extracellular plaques and within the vasculature. Together, these results demonstrate the deposition of phosphorylated Aβ species in DS brains, further supporting the similarity of Aβ deposition in AD and DS. Thus, the detection of phosphorylated and other modified Aβ species could contribute to the understanding and dissection of the complexity in the age-related and spatiotemporal deposition of Aβ variants in AD and DS as well as in distinct mouse models.

## Introduction

Depositions of amyloid-β (Aβ) peptides as senile plaques in the brain parenchyma and in the walls of cerebral blood vessels are common neuropathological features of the Alzheimer’s disease (AD) [1–3]. Aβ peptides derive from the proteolytic processing of the amyloid precursor protein (APP) by proteases called β- and γ-secretase [4–6]. In addition to the generation and deposition of well-characterized Aβ40 and Aβ42 amino acid length variants, recent studies also showed the occurrence of several N- and C-terminally truncated or elongated Aβ species that result from alterations in the cleavage by β- and γ-secretase or alternative processing by other proteases [7–13]. Additional heterogeneity in Aβ peptides comes from a number of post-translational modifications that are also found in characteristic Aβ deposits in parenchymal extracellular plaques and cerebral amyloid angiopathy (CAA) [14–22]. N- and C-terminal truncated or elongated species as well as post-translationally modified Aβ have specific characteristics in aggregation and biostability [23–28].

Down syndrome is a genetic disorder caused by an extra copy of chromosome 21, and characterized by specific facial and neurological features. People with DS also have an increased risk of developing early onset AD [29–31]. Interestingly the gene encoding APP is localized within a region of chromosome 21 that is critical for DS, and the triplication of the *APP* gene results in elevated levels of Aβ peptides that form amyloid plaques at least two decades prior to the onset of the clinical AD-like symptoms [31–33]. DS brains demonstrate Aβ plaques already at 12–30 year of age, principally in the form of diffuse Aβ plaques, the type of early Aβ pathology also seen at pre-clinical (i.e., pathological aging) and prodromal stages of sporadic AD [30, 34–36]. In DS subjects, aged > 40 years, levels of cortical Aβ deposition are similar to those observed in sporadic, late onset AD and demonstrate cored neuritic plaques, which are of neuropathological diagnostic significance in AD [29–31].Notably, autopsy studies of DS brain show the occurrence of isomerized, racemized, truncated, pyroglutamate and oxidized Aβ, indicating the accumulation of post-translationally modified Aβ variants as reviewed previously  [30].

We recently showed that Aβ undergoes phosphorylation at serine residue 8, which affects its conformation, aggregation, neurotoxicity and proteolytic degradation [18, 25, 27, 37, 38]. Phosphorylated Ser8-Aβ (pSer8Aβ) occurs in vivo in the brains of human AD patients, non-human primates and canines [39–42]. Notably, the detection of pSer8Aβ, together with pyroglutamate modified Aβ in brain sections or brain extracts has recently been explored to establish a staging system for AD pathology based on the sequential deposition of these modified Aβ variants during the pathogenesis of AD [42]. While pyroglutamate modified Aβ could already be detected in the pre-clinical stage of AD, pSer8Aβ species occur selectively in the clinical phase of AD. These findings support a role of modified Aβ species in the pathobiology of AD.

Murine models are crucial for the advancement of our understanding of Aβ deposition in AD. Several transgenic mouse models have been generated that overexpress APP with or without familial Alzheimer disease (FAD) mutations or combinations of human mutant APP, PS, and tau transgenes that reflect certain aspects of human AD [43–47]. These AD mouse models are immensely important for the investigation of AD related pathophysiological processes.

Here, we analysed the occurrence of pSer8Aβ in the brains of DS cases, and in different single, double and triple transgenic mouse models of AD. Our data demonstrate the deposition of phosphorylated Aβ in DS cases thereby further supporting the similarity of Aβ lesions in DS and AD. pSer8Aβ was also found in all transgenic mouse models analysed and showed characteristic age-dependent, and spatiotemporal deposition. Together, these results indicate that deposition of pSer8Aβ is a common feature of pathological Aβ deposition in the human brain, and could be further explored to dissect the composition of Aβ lesions in different transgenic mouse models.

## Material and methods

### Human subjects

We used phosphorylation-state specific monoclonal antibodies to characterize the deposition of phosphorylated (pSer8Aβ) and non-phosphorylated (npAβ) variants of Aβ in 3 DS (46, 47 and 55 years), 12 sporadic AD (average, 86 ± 7 years) and 8 non-demented aged control (AC; average, 67 ± 10 years) brains (Table 1). Human brain tissue was collected at the time of autopsy, having obtained prior consent from the next of kin and following protocols approved by the Partners Human Research Committee at Brigham and Women’s Hospital (Boston, MA, USA). Human cortical and hippocampal brain blocks of AC, AD and DS were fixed in 10% neutral buffered formalin for two hours (brief fixation) with the exception of one DS brain (47 years old) that was fixed for about 1 month (routine fixation) before undergoing washing in PBS and paraffin processing. Human subject information including age, gender, and brain region examined (frontal, occipital, parietal, temporal/hippocampal, hippocampal) and semi-quantitative staining results for this study are summarized in Table 1.

**Table 1:** Examination summary of aged control (AC), Alzheimer’s disease (AD) and Down syndrome (DS) brains

Cases	Age (years)	Gender	Brain region examined	82E1 (Aβ1-x)	7H3D6 (npAβ)	1E4E11 (pSerAβ)	PMI (hours)	Brain weight (grams)	Cause of Death	Braak Stage
**Aged Control (AC)**										
AC-1	72	Female	F	–	–	–	NA	1300	Cardiac arrest	NA
			HC	+	+	(+)				
AC-2	53	Male	F	–	–	–	9	1500	Cardiac arrest	NA
			HC	+	–	–				
AC-3	60	Female	F	+++	–	–	11	1150	Cardiac arrest; Sepsis	NA
			O	+++	–	–				
AC-4	64	Male	HC	+++	+	–	9.5	1380	Congestive heart failure	NA
AC-5	70	Male	F	++	+	(+)	17	1500	Mesothelia carcinoma	NA
			HC	+++	+	+				
AC-6	60	Female	F	++	–	–	3.5	1380	COPD; AS	NA
			HC	++	–	–				
AC-7	87	Female	F	+++	+	(+)	NA	NA	Choking	NA
			T/HC	–	–	–				
AC-8	74	Male	F	++	–	–	NA	1400	Dead on Arrival	NA
**Alzheimer’s Disease (AD)**										
AD-1	82	Female	F	++++	+	++	16	900	Pneumonia	NA
			HC	+++	+	++				
AD-2	79	Female	F	+++	++	+	13	1040	AD; AS	NA
			HC	+++	+	++				
AD-3	91	Female	T	++	+	–	3.5	1370	Pneumonia; AD; AS; Lacunar infarct	III -IV
			HC	+++	+	++				
AD-4	71	Male	F	+++	+	++	36	NA	AD; AS	NA
			HC	+++	+	++				
AD-5	84	Female	F	+++	–	++	NA	1000	AD; AS; Binswanger	NA
			T	++	++	–				
AD-6	92	Female	HC	+++	+	++	7	1210	AD; AS; Infarct	NA
AD-7	78	Male	F	+++	+	++	18	1200	AD; AS	V-VI
			HC	++	–	+				
AD-8	96	Female	F	++	++	++	21	1050	AD; AS; Infarct	NA
			HC	+++	+	++				
AD-9	78	Male	P	+	++	+	NA	1180	AD; Subdural hematoma	V-VI
			T/HC	+++	+++	+				
AD-10	88	Female	P	+++	+	+	24	1220	AD; AS	
			HC F	++++	++	+++				
AD-11	84	Female	F	+++	–	++	16.5	1110	AD; AS; DLBD	NA
			HC	+++	+	++				
AD-12	88	Female	F	+++	+	+	24	1050	AD; AS; Infarct	V-VI
			HC	++	++	+				
**Down syndrome (DS)**										
DS-1	47	Male	F	++++	+	++	34	910	Glioblastoma; AD	NA
			T/HC	++++	+	++				
DS-2	55	Male	F	++++	++	++	NA	1040	Pneumonia; AD	NA
			T	++++	++	+++				
DS-3	46	Female	T/HC	+++	++	++	18	870	Pneumonia; AD	NA

The following semi-quantitative scoring criteria were used: –, no staining; (+), 1–10 plaques; +, 11–30 plaques; ++, 31–50 plaques; +++, 51–100 plaques, and ++++, > 100 plaques per cm^2^. *F* frontal; *HC* hippocampal; *T* temporal; *O* occipital; *P* parietal; *DLBD* Diffuse Lewy Body Disease; *COPD* Chronic Obstructive Pulmonary Disease; *AS* Arteriolar sclerosis; *NA* not available

### Transgenic AD mouse models

Mouse brain tissue was obtained from six transgenic mouse lines that are commonly used as AD models, namely J20 [48], hAPP751 [49], TgSwDI [50], APP/PS1ΔE9 [51], PS/APP [52], and 3xTg-AD [53] (Table 2). Hemibrains were fixed in 10% formalin or 4% paraformaldehyde for 2 to 24 h before being processed for paraffin embedding and then sectioned at 10 μm. All use of mice at Brigham and Women’s Hospital was approved by the Harvard Medical Area Standing Committee on animals and was in compliance with state and federal regulations.

**Table 2:** Transgenic AD-like mouse models in which pSer8Aβ and npAβ deposition has been analyzed. APP, Amyloid precursor protein; PS1, Presenilin-1; h-human; m-mouse; Swe, Swedish; Ind, Indiana; Lon, London; PSEN1, Presenilin-1; KI, Knock-in; Tg, Transgenic; Ref, Reference

Mouse model	Transgene	Ref.	Transgenic promoter	Age (months)	Amyloid plaques		CAA lesions	
					npAβ	pSer8Aβ	npAβ	pSer8Aβ
**APP Transgenics:**								
J20	hAPP770	48	*PDGFβ*	4 (*n* = 4)	+	–	No	No
	(K670N/M671L; V717F)			8 (n = 4)	++	+	Yes	Yes
	Swe; Ind mutation			16 (*n* = 3)	++++	+++	Yes	Yes
hAPP751	hAPP751	49	mouse *Thy-1*	14 (*n* = 2)	++++	+++	Yes	Yes
	(K670N/M671L; V717I)							
	Swe; Lon mutation							
TgSwDI	hAPP770	50	mouse *Thy-1*	3 (n = 4)	+	+	Yes	Yes
	(K670N/M671L; E693Q; D694N)			6 (n = 2)	++	+	Yes	Yes
				12 (n = 4)	+++	++	Yes	Yes
	Swe; Dutch; Iowa mutation			24 (n = 4)	++++	+++	Yes	Yes
**APP/PS1 Transgenics:**								
APP/PS1ΔE9	m/hAPP695	51	mouse *PrnP*	6 (n = 4)	++	+	Yes	Yes
	(K595N/M596L);			17 (n = 3)	+++	++	Yes	Yes
	hPS1 deletion of exon 9			24 (n = 4)	++++	+++	Yes	Yes
	Swe; PSEN1ΔE9							
PS/APP	hAPP695 hPS1 (M146L) Swe; PS1M146L	52	Hamster *PrnP* (APP) *PDGFβ* (PS1)	18 (*n* = 5)	++++	+++	Yes	Yes
**APP/PS1/Tau Transgenics:**								
3xTg-AD	hAPP695 (K670N/M671L); hPS1 (M146V); TauP301L Swe;PS1M146V;MAPT4R0N (P301L)	53	mouse *Thy-1.2* (APP, Tau) PS1 KI	5 (n = 4)	++	+	No	No
				14 (n = 3)	+++	++	Yes	Yes
				27 (n = 4)	++++	+++	Yes	Yes

The following semi-quantitative scoring criteria were used: -, no plaque staining, +, 1–5 plaques; ++, 6–10 plaques; +++, 11–100 plaques; ++++, > 100 plaques. Abbreviations: *Ref* References; *Swe* Swedish; *Ind* Indiana; *Lon* London; *PS1* presenelin-1, *MAPT*, microtubule-associated protein tau; *PDGFβ* platelet-derived growth factor B-chain; *PrnP* prion protein; KI, knock-in

### Immunohistochemistry

Immunohistochemistry was performed on 8–10 μm thick sections of hippocampus and cortex as previously described [20, 41], using three different primary antibodies. In brief, human brain sections were deparaffinized in two changes of Histo-Clear (National Diagnostics, Atlanta, GA) and rehydrated in graded ethanol solutions. Endogenous peroxidase activity was quenched with 0.3% H_2_O_2_ in methanol for 10 min. All paraffin sections were pretreated with 88% formic acid for 8 min to increase recognition of antigen binding sites. Sections were subsequently washed with water for 10 min and incubated with primary antibody overnight at 4 °C. After incubation for 30 min at room temperature with a biotinylated secondary antibody (Vector Laboratories). Immunoreactivity was visualized with the VECTASTAIN Elite horseradish peroxidase ABC kit (Vector Laboratories) and DAB (Sigma-Aldrich) as chromogen.

When using mouse monoclonal antibodies on mouse brain sections, a Mouse on Mouse (M.O.M.) kit (Vector Laboratories) was used to inhibit nonspecific background staining. The mouse monoclonal antibody (mAb) 82E1 (dilution 1:500; Immuno-Biological Laboratories, Japan) recognizes Aβ peptides starting at aspartic acid 1 (Aβ1-x). The murine 1E4E11 mAb (dilution 1:500 - pSer8Aβ) is reactive to Aβ peptides phosphorylated at Ser8, and rat 7H3D6 mAb (dilution 1:500 - npAβ) specifically recognizes Aβ peptide with Ser8 in a non-phosphorylated state [37]. The specificity of the phosphorylation-state specific antibodies 1E4E11 and 7H3D6 was demonstrated previously by pre-adsorption with synthetic Aβ peptides with Ser8 in phosphorylated or non-phosphorylated state by western immunoblotting and immunohistochemistry and by staining with secondary antibodies alone [37, 39]. Semi-quantitative analysis was performed by scoring immunoreactivity of each antibody by a person blinded to the diagnosis associated with each brain sample. Sets of immunostained serial sections from AC, AD and DS cases were photographed, and the number of only distinct plaques, both the diffuse and compact types within the region of interest was analyzed using ImageJ image processing and analysis software (National Institutes of Health, Bethesda, USA). Gray scale thresholding was used to identify positively stained structures from background staining. Operator editing was used to remove staining artifacts. For human brain tissues, the number of npAβ and pSer8Aβ-immunopositive plaques in specific brain regions was semi-quantitavely scored as: –, no staining; (+), 1–10 plaques; +, 11–30 plaques; ++, 31–50 plaques; +++, 51–100 plaques, and ++++, > 100 plaques per cm^2^. For transgenic mouse samples, semi-quantitative analysis of total npAβ and pSer8Aβ immunoreactivity was performed using Bioquant image analysis software, which allows identification of objects based on thresholding of the optical density to identify Aβ deposits. The threshold of detection was held constant during analysis, and the imager was blinded to the study. 4–6 sections per mouse were analysed by counting the number of npAβ and pSer8Aβ positive plaques in the entire hippocampus and cortex. The analysed areas were kept constant for all sections. Plaque scoring of each group is determined by calculating the sum of individual scores divided by the number of animals per group. Scoring key: –, no plaque staining; +, 1–5 plaques; ++, 6–10 plaques; +++, 11–100 plaques; ++++, > 100 plaques per area. Images were captured in a single session using a constant threshold under a Nikon Eclipse E400 microscope.

## Results

**Fig. 1:**
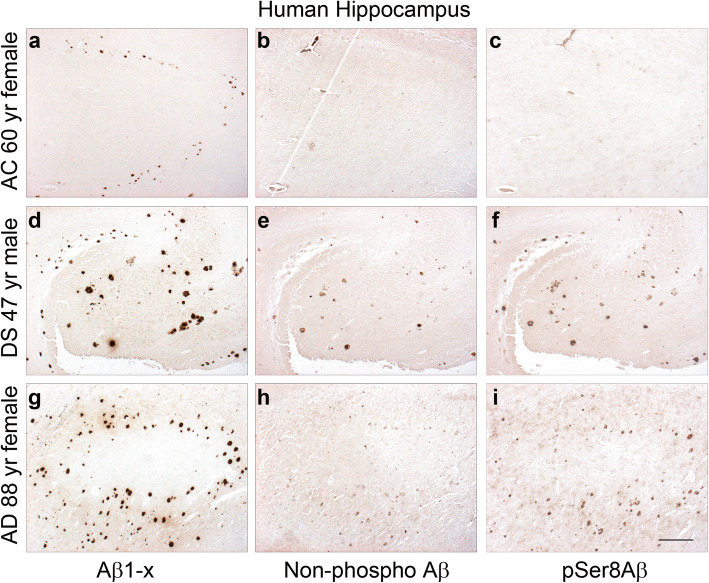
Immunohistochemical detection of pSer8Aβ peptides in DS and AD brains. Immunohistochemical staining was performed on adjacent serial paraffin sections with monoclonal antibodies 82E1 (Aβ1-x), 7H3D6 (non-phoshoAβ) and 1E4E11 (pSer8Aβ). Asp-1 Aβ (Aβ1-x), npAβ and pSer8Aβ species colocalized in DS (**d**-**f**) and AD (g-i) hippocampus. pSer8Aβ and npAβ were detected in extracellular Aβ plaques in DS and AD brains. AC brains showed some immunoreactivity with 82E1antibody (**a**), but very limited if any reactivity with antibodies 7H3D6 (**b**) and 1E4E11 (**c**). Scale bar = 100 μM

Human hippocampal brain sections were stained with monoclonal antibodies specifically recognizing the N-terminus of Aβ starting at amino acid Asp1 (Aβ1-x) (Fig. 1a, d and g), with monoclonal antibodies specifically recognizing Aβ with Ser8 in phosphorylated (pSer8Aβ) (Fig. 1c, f and i, supplementary Fig. 1) or non-phosphorylated state (non-phospho Aβ; npAβ) (Fig. 1b, e and h, supplementary Fig. 1) to assess Aβ deposition in 3 DS, 12 AD and 8 AC brains. Results of a semi-quantitative analysis of Aβ1-x, pSer8Aβ and npAβ deposition in extracellular plaques are summarized in Table 1. Figure 1 shows immunoreactivity of Aβ1-x, npAβ and pSer8Aβ deposits in hippocampus of a 60-year-old female AC (Fig. 1a-c), a 47- year-old male DS individual (Fig. 1d-f) and an 88-year-old female AD patient (Fig. 1g-i). pSer8Aβ was present in extracellular Aβ plaques in all three DS cases, and all AD cases (Table 1).

The 3 DS cases had abundant deposits of Aβ1-x also containing pSer8Aβ and npAβ peptides (Table 1, Fig. 1d-f). In all 3 DS brains, extracellular plaques were found in the hippocampus, the frontal cortex as well as in the temporal cortex. Overall, there was an overlap in the immunoreactivity for pSer8Aβ and Aβ variants starting with Asp1 of the Aβ sequence (Aβ1-x; Fig. 1d and f). Immunostaining with 7H3D6 mAb detected fewer plaques in DS brains (Fig. 1e; Table 1). It is important to note that the 7H3D6 antibody does not detect Aβ species modified by N-terminal truncation (Aβ3–42), pyroglutamate formation (pyroGluAβ3–42), nitration (3NTyr10-Aβ) or phosphorylation at Ser8 (pSer8Aβ) (Supplementary Fig. 1). It is therefore possible that beside phosphorylation, the presence of other modifications and truncation of Aβ also contributes to the decreased reactivity of the monoclonal antibody 7H3D6 in these samples. Indeed, abundant levels of amino-terminally modified and truncated Aβ peptides have been detected in DS brains previously [30, 35, 36, 54, 55].

All AD brains showed robust Aβ deposits (Table 1), that were also detected by the pSer8Aβ specific antibody 1E4E11 (Fig. 1g, i). Aβ plaques in AD cases were predominantly found in the frontal cortex and contained both pSer8Aβ and npAβ variants (Table 1). All 8 control (AC) brains showed Aβ1-x positive plaques at variable levels (Table 1, Fig. 1a-c). In contrast, pSer8Aβ positive plaques were absent in 4 and only very weakly detected in the other 4 control brains, consistent with the occurrence of pSer8Aβ selectively in the clinical phase of AD [39, 42]. These results indicate that deposition of pSer8Aβ is characteristic for symptomatic AD as well as DS cases.

**Fig. 2:**
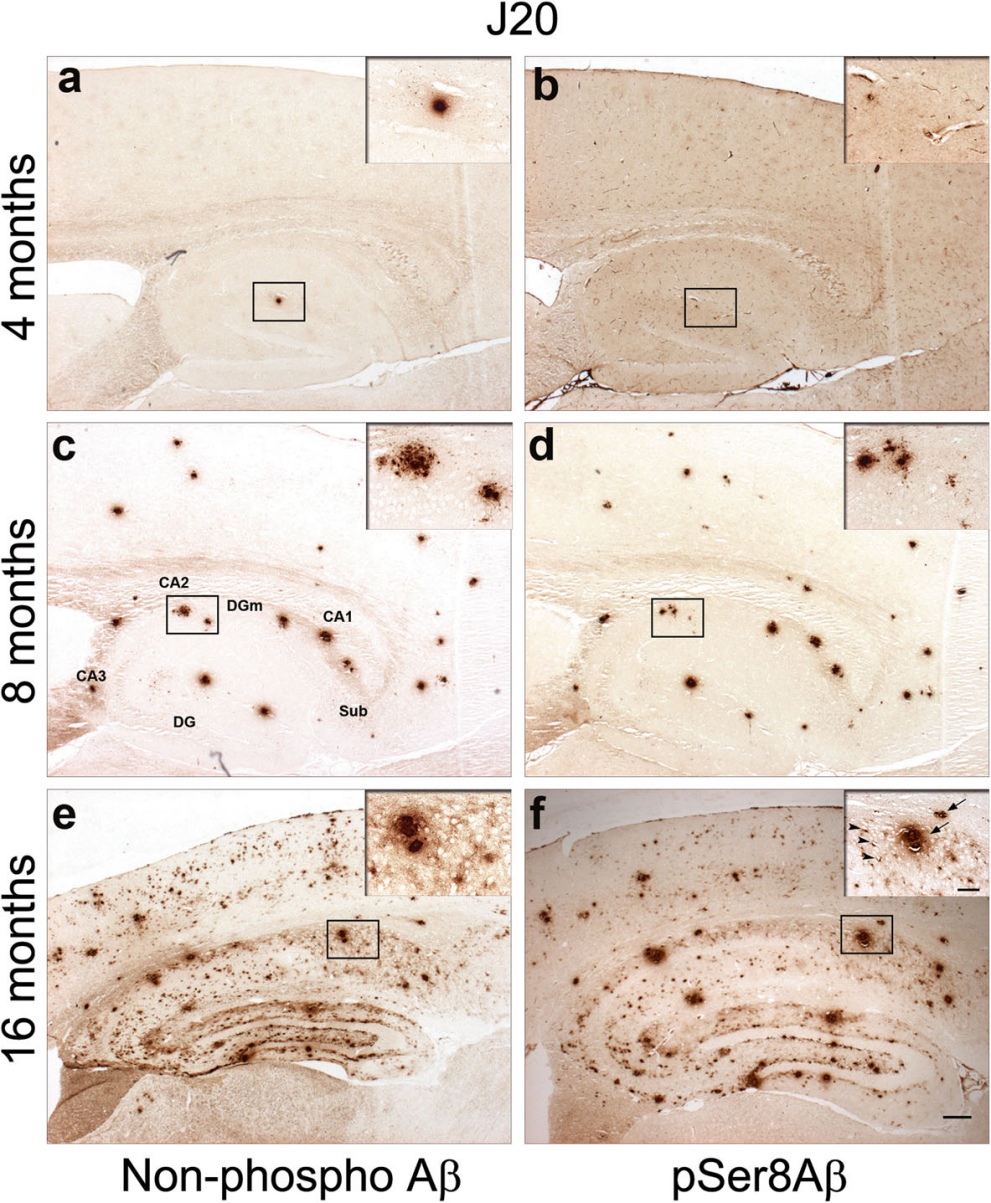
Age-dependent deposition of cerebral npAβ and pSer8Aβ in J20 mice. Brain sections of 4, 8, and 16 month-old mice were immunolabeled with npAβ (**a**, **c** and **e**) and pSer8Aβ-specific (**b**, **d** and **f**) antibodies. npAβ deposits were detected initially in the hippocampus at 4 months (**a**). A faint reactivity of pSer8Aβ was seen in extracellular amyloid deposits and in some vessels in the hippocampus (**b**; inset). By 8 months, npAβ (**c**) and pSer8Aβ deposition (**d**) extended to the neocortex as compact and diffuse deposits. By 16 months, more intense and abundant npAβ (**e**) and pSer8Aβ-immunoreactivity (**f**) was observed in extracellular plaques (arrows) and blood vessels (arrowheads) affected by CAA (inset in panel **f**). Sub, subiculum; DG, dentate gyrus; DGm, dentate gyrus molecular layer. Scale bar = 200 μm. Insets show the magnified areas indicated by the boxes. Scale bar = 50 μm

We also investigated several established transgenic AD mouse models for the deposition of pSer8Aβ and npAβ peptides by immunohistochemistry. Details on the different transgenes and semi-quantitative analysis of pSer8Aβ and npAβ immunoreactivity in each mouse model are summarized in Table 2. The different mouse models were grouped by their AD related transgenes. The J20 [48], hAPP751 [49] and TgSwDI [50] models, express only APP transgenes with different combined familial AD mutations (Table 2). The J20 mouse model expresses the hAPP770 variant with two mutations linked to familial AD (the Swedish and Indiana mutations) under control of the *PDGFβ* promotor. Aβ deposition in J20 mice starts between 4 and 5 months of age as diffuse Aβ plaques in the hippocampus. By 8–10 months, these mice show progressive and widespread Aβ deposition [48]. We examined the deposition of pSer8Aβ and npAβ peptides at 4, 8 and 16 months of age. Only a very modest level of npAβ immunoreactivity was observed in two of the four J20 mice at 4 months of age, exclusively detected in the hippocampus (Fig. 2a, Table 2). At this age, very faint reactivity of pSer8Aβ appeared in few extracellular amyloid deposits and in some vessels (Fig. 2b, inset). At 8 months of age, J20 mice displayed more extracellular plaques. At this age most of the npAβ positive plaques in the hippocampus and cortex also contained pSer8Aβ (Fig. 2c and d). At 16 months, J20 mice had substantial deposition of npAβ and pSer8Aβ in the neocortex, hippocampus, and subiculum (Fig. 2e and f, Table 2). Diffuse npAβ and pSer8Aβ immunoreactivity was additionally present along the molecular layer of the dentate gyrus (DGm). pSer8Aβ and npAβ deposits were also detected in leptomeningeal and parenchymal blood vessels at 16 months of age (Fig. 2e and f, and Supplementary Fig. 2a-d).

A very similar distribution and high abundance of npAβ and pSer8Aβ was also observed in a different mouse model that overexpresses the human APP751 isoform containing the Swedish and London mutations under control of the *Thy-1* promotor [49]. Amyloid plaques in hAPP751 mice start to develop at 3–4 months of age in the frontal cortex, and at 5–7 months, dense amyloid deposits are observed in the hippocampus, thalamus, and olfactory region [49]. At 14 months of age, brains showed strong immunoreactivity for npAβ and pSer8Aβ in diffuse and compact plaques throughout the entire hippocampus, the neocortex and thalamus, as well as in leptomeningeal and parenchymal blood vessels (Supplementary Fig. 2e-h, Table 2).

**Fig. 3:**
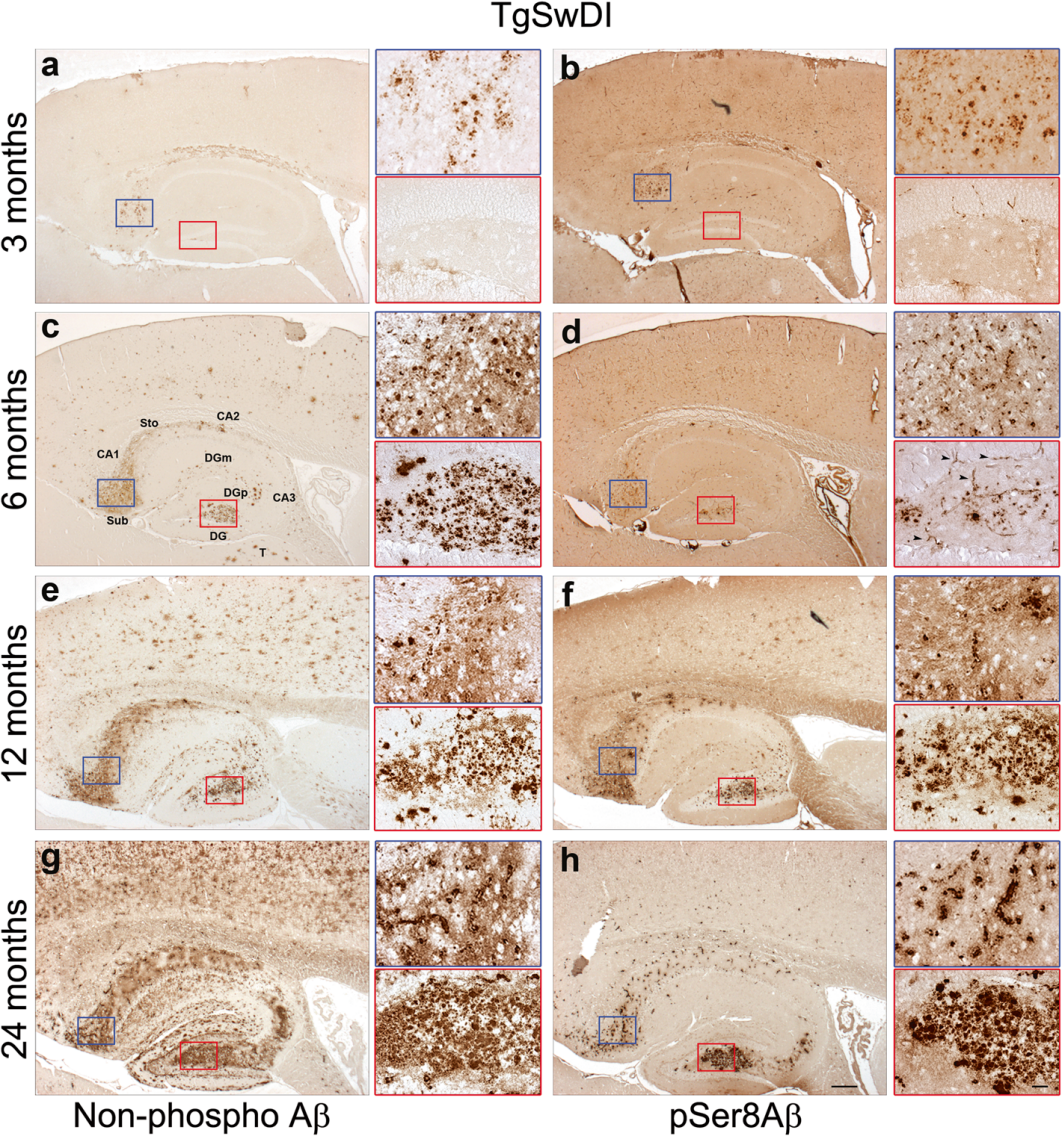
pSer8Aβ deposition in TgSwDI mice at different ages in CA1 region and molecular layer of dentate gyrus (DG). Analysis of consecutive sections using npAβ and pSer8Aβ antibodies in TgSwDI mice at 3, 6, 12 and 24 months of age. At 3 months, minute amounts of npAβ and pSer8Aβ reactivity were seen in the subiculum (**a**, **b**). At 6 months, deposition of npAβ and pSer8Aβ increased and extended to the CA1 region and dentate gyrus (**c**, **d**). By 12 months, both npAβ and pSer8Aβ were increased in the vasculature between the pyramidal neurons and in the stratum oriens (**e**, **f**). By 24 months, abundant CAA was observed by intense npAβ immunoreactivity that colocalized almost completely with pSer8Aβ (**g**, **h**). Sub, subiculum; Sto, stratum oriens; DG, dentate gyrus; DGm, dentate gyrus molecular layer; DGp, dentate gyrus polymorphic layer; T, thalamus. Scale bar = 200 μm. Enlarged images (insets) in the subiculum and dentate gyrus are indicated by blue and red boxes, respectively. Scale bar = 50 μm

We next analysed the age-dependent deposition of npAβ and pSer8Aβ in brains of TgSwDI mice that express the hAPP770 variant harboring three different APP mutations (Swedish; Dutchand Iowa) (Table 2). Notably, the Aβ variants with Dutch and Iowa mutations are vasculotropic and associated with CAA [50]. As shown in Fig. 3, the TgSwDI mouse model had some of the earliest and most abundant cerebral pSer8Aβ deposition, seen as extracellular diffuse plaques and vascular deposits, predominantly in the subiculum region, at 3 months of age that increased with age (Fig. 3a and b). At 6 months of age, increased Aβ extracellular deposits were observed together with abundant Aβ accumulation in and around blood vessels (Fig. 3c and d). At 6 months of age, npAβ deposition was detected in the thalamus, stratum oriens, CA1, DGm, and dentate gyrus polymorphic layers (DGpl), and concomitantly increased in the subiculum (Fig. 3c). pSer8Aβ immunoreactivity was observed in a subset of npAβ positive plaques in the subiculum, thalamus, and stratum oriens, and CA1 hippocampal region of the brain (Fig. 3d). At this age, only modest amounts of npAβ and pSer8Aβ deposition were observed in the neocortex. By 12 months of age, Aβ deposits were observed throughout the brain (Fig. 3e and f). pSer8Aβ deposition was increased in the aforementioned regions and found in a subset of npAβ deposits in the hippocampal CA2 region, DGm, and neocortex (Fig. 3e and f). At 24 months of age, pSer8Aβ reactivity was observed in a subset of npAβ-positive diffuse plaque-like and vascular Aβ deposits (Fig. 3h) that were far more abundant in the hippocampus (specifically in the CA1, CA2, CA3, DGm, DGpl, stratum oriens), the thalamus, and neocortex (Fig. 3g) compared to those found in younger mice.

**Fig. 4:**
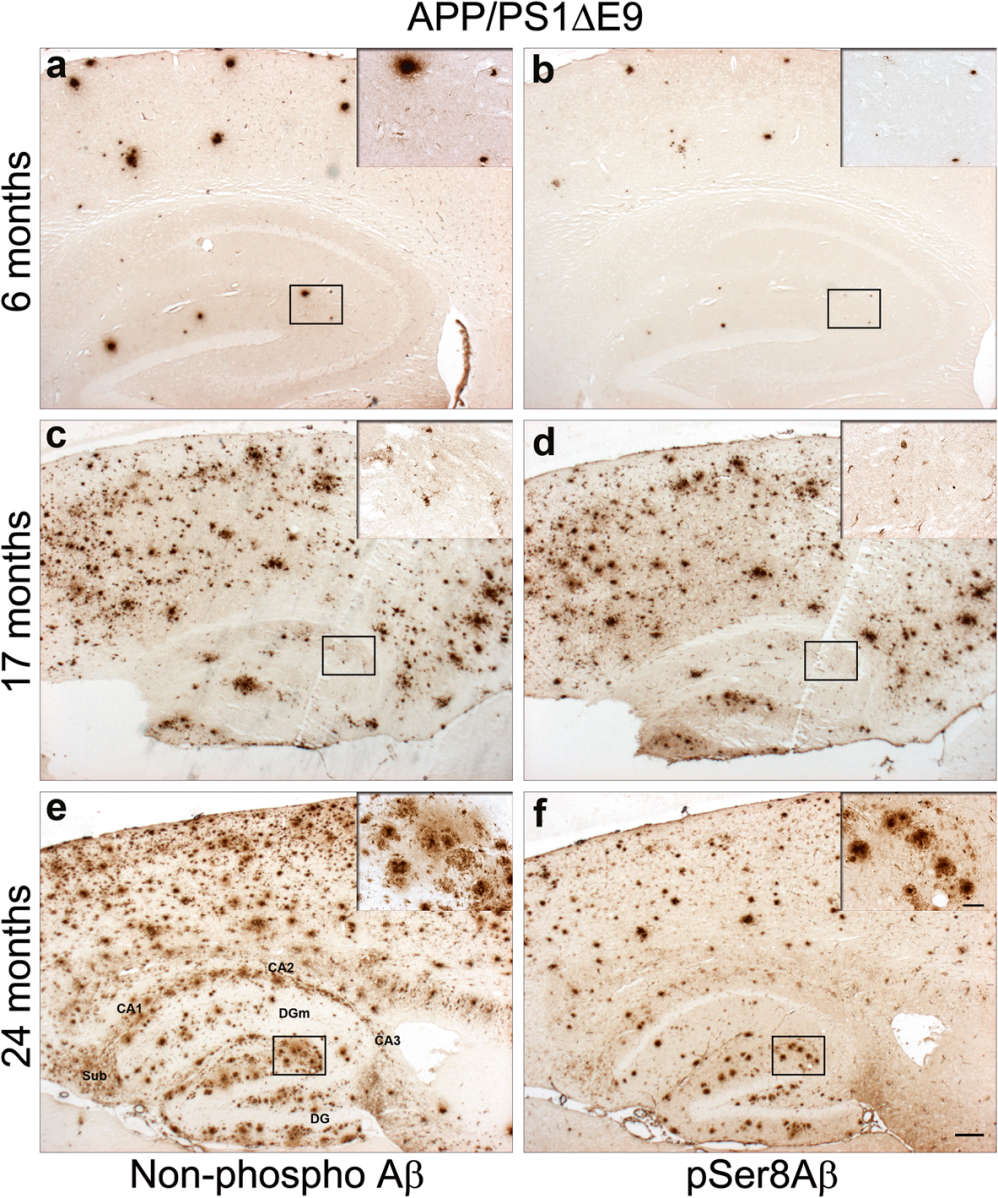
pSer8Aβ deposition in APP/PS1ΔE9 mice at different ages. Detection of npAβ (**a**, **c** and **e**) and pSer8Aβ (**b**, **d** and **f**) in consecutive sections of APP/PS1ΔE9 mice at 6, 17 and 24 months revealed pSer8Aβ-positive parenchymal and vascular immunoreactivity, which was in most cases less abundant than npAβ-immunoreactivity. Scale bar = 200 μm. Sub, subiculum; DG, dentate gyrus; DGm, dentate gyrus molecular layer. Insets show the magnified areas indicated by the boxes. Scale bar = 50 μm

In addition, we analysed two double transgenic mouse models carrying FAD associated mutations in APP and PS1 (APPswe/PS1ΔE9 and PS/APP mice). The APPswe/PS1ΔE9 mice, harboring the FAD-associated Swedish mutation in APP and the exon 9 deletion in PS1 [51, 56]. This model develops plaque deposition by 6 months of age in the hippocampus and cortex. At this age, we detected npAβ positive plaques in the hippocampus and neocortex (Fig. 4a). Notably, pSer8Aβ immunoreactivity was only observed in a small subset of compact plaques in the hippocampus as well as in the neocortex in two of four mice, but not elsewhere at this age (Fig. 4b and Table 2). By 17 months, npAβ-immunopositive extracellular plaques, and vascular deposits had increased dramatically in the neocortex and hippocampus (Fig. 4c). Interestingly, APPswe/PS1ΔE9 mice at 17 months displayed robust pSer8Aβ immunoreactivity in diffuse and compact deposits in the neocortex as well as in the parenchymal blood vessels and compact deposits in the hippocampus (Fig. 4d). By 24 months, abundant npAβ immunoreactivity was noted in the hippocampus and neocortex (Fig. 4e). Most compacted plaques in the hippocampus and a subset of neocortical plaques were pSer8Aβ-positive (Fig. 4f). Strong npAβ immunoreactivity was observed in leptomeningeal blood vessels, and plaques in the molecular layer of the hippocampus that often colocalized with pSer8Aβ immunoreactivity (Fig. 4e and f).

Another double transgenic mouse model with overexpression of hAPPswe and a PS1 FAD-associated mutation was generated previously by crossing APPswe (Tg2576) transgenic mice with mutant *PSEN1* (PS1M146L) expressing transgenic mice [52]. These double transgenic PS/APP mice develop Aβ deposits in the cortex and hippocampus by 3 months of age that increase with aging [57]. Brains of 18 month-old mice contained abundant npAβ and pSer8Aβ positive deposits (Supplementary Fig. 2i and j). npAβ appeared as extracellular plaques throughout the cortex, hippocampus, and thalamus (Supplementary Fig. 2i and k). pSer8Aβ reactivity is observed in a subset of npAβ-positive extracellular plaques (Supplementary Fig. 2j and l). npAβ and pSer8Aβ reactivity was also noted in vascular deposits in the parenchyma pial surface and leptomeninges (Supplementary Fig. 2i-l).

Furthermore, we analysed the brains of triple transgenic (3xTg-AD) mice possessing the Swedish APP, the PS1 M145V, and the Tau P301L mutations [53]. This model, in addition to the deposition of Aβ in extracellular plaques and the formation of intraneuronal tau aggregates, also shows prominent accumulation of intraneuronal Aβ immunoreactivity [53]. Examination of cortical brain sections at different ages (Table 2), revealed that npAβ and pSer8Aβ immunoreactivity increased with age as intense compact deposits in different brain regions, with npAβ deposition being more abundant in extracellular deposits throughout the hippocampus and cortex (Supplementary Fig. 2 m and n). Abundant intraneuronal npAβ accumulation also contained pSer8Aβ immunoreactivity in neocortical regions (Supplementary Fig. 2o and p). The antibodies 7H3D6 and 1E4E11 used to detect npAβ and pSer8Aβ do not crossreact with the full-length APP or its C-terminal fragments [37, 40, 42]. Thus, these data further support the pronounced accumulation of intracellular Aβ in this triple transgenic mouse model [53, 58–60].

In summary, pSer8Aβ is present in all APP-overexpressing models examined in this study, and largely shows co-deposition with npAβ in extracellular plaques, blood vessels, and intraneuronal Aβ aggregates, despite the specific spatiotemporal pattern of the deposits observed in the different transgenic models.

## Discussion

Using phosphorylation-state specific monoclonal antibodies, we characterized Aβ pathology in human AD, AC and DS brains, and in several transgenic mouse models. Consistent with our previous findings [18, 39, 42], npAβ and pSer8Aβ deposits were present in extracellular plaques of AD brains, while AC brains contained much lower amounts of npAβ and even less pSer8Aβ in a subset of cases. Notably, all three DS brains also revealed the presence of npAβ and pSer8Aβ in extracellular plaques. Interestingly, immunostaining with an antibody (7H3D6) that is highly specific for N-terminally unmodified Aβ species (Supplementary Fig. 1) [37], resulted in only weak staining in individual AD and DS brains (Table 1). Given the high reactivity with the antibody 82E1, which specifically recognizes Aβ starting at Asp1 and does not detect N-terminally truncated or pyroGlu-modified Aβ species, the data could suggest that Aβ species starting at Asp1 contain post-translational modifications, including phosphorylation at Ser8 or nitration of Tyr10. However, specific characteristics of the different antibodies regarding the conformation of aggregated Aβ in the deposits or the processing of brain samples could also affect the detection of the different Aβ species. It is important to note that, in contrast to most generic Aβ antibodies used to detect Aβ pathology, the monoclonal phosphorylation-state specific Aβ antibodies used in the present study specifically detect Aβ peptides without cross-reactivity with full-length APP and thus, allow unambiguous detection of extracellular and intracellular Aβ aggregates.

In addition to the well characterized Aβ1–40/42 species, N- and C-terminally truncated or post-translationally modified forms of Aβ peptides also exist in AD brains and might contribute to neurodegeneration [12, 16, 21–24, 61, 62]. Thus, detailed analyses of Aβ species that constitute amyloid deposits in AD is of major interest. The DS cases demonstrated pSer8Aβ reactivity in both diffuse and compact plaques that in part overlap with the immunoreactivity of npAβ and Aβ1-x variants starting with Asp1. Together with the previous demonstration of truncated Aβ and pyroGlu-modified Aβ peptides in AD and DS brains [20, 21, 23, 30, 36, 42, 61, 62], the detection of pSer8Aβ immunoreactivity in DS brains shown here provides strong evidence for similar mechanisms of amyloidogenesis and composition of plaques in DS and AD. Thus, it will be interesting to further investigate how the pSer8Aβ species will affect senile plaque formation and other features of brain pathology in DS and AD cases. It also remains to be elucidated, which mechanisms lead to the accumulation of pSer8Aβ in DS and AD. Altered expression of protein kinases and phosphatases has been described for DS and AD [63–66]. Our previous studies showed that protein kinase A (PKA) is capable to efficiently phosphorylate Aβ in vitro and cultured primary neurons [18]. However, the relative contribution of PKA or other kinases to the phosphorylation of Aβ in human brain is not known. It is also possible that a decrease in phosphatase activities could contribute to the accumulation of phosphorylated Aβ species during the pathogenesis of AD and DS.

Studies in vitro, with cultured neurons, and Drosophila models showed that phosphorylation promotes the aggregation and increases toxicity of Aβ [18, 26]. However, in human brains, pSer8Aβ deposition is detected predominantly in the symptomatic phase of AD [40, 42], when a considerable degree of synapse loss has already occurred. Thus, it will be interesting to investigate whether pSer8Aβ and other Aβ variants initiate neuronal damage as soluble species. Indeed, soluble oligomeric Aβ species are considered to exert higher neurotoxicity as compared to fibrillar assemblies in extracellular plaques [67–69].

We also demonstrate the deposition of pSer8Aβ in a variety of AD Tg mouse models. Mouse models of AD that accurately recapitulate major characteristics of Aβ pathology are critical for better understanding molecular mechanisms of pathogenesis, and to assess novel therapeutics in preclinical studies [43–47]. Most of these models have been generated by transgenic overexpression of the gene encoding human APP (alone or in combination with human *PSEN1* or human *MAPT*), and present with progressive accumulation of Aβ in form of extracellular plaques and neurofibrillary tangles. Some of these Tg mouse models develop CAA to various degrees, which allow studying the effect of Aβ accumulation on vascular function [46, 70, 71]. These transgenic models use various promoters to drive transgene expression in different genetic back grounds (Table 2). Interestingly, such mouse models showed that neuronal Aβ generation could drive CAA [72], and impaired Aβ clearance seems to enhance CAA [73]. Our study supports a critical role of Aβ generated by neurons in the formation of CAA, as abundant CAA was observed in transgenic mice with APP expression controlled by the neuron-specific *Thy-1, Prnp* or *PDGFβ* promoters (Table 2), although minor contribution of Aβ generated by additional brain cells cannot be excluded. Early onset (~ 3 months of age) and progressive accumulation of CAA is observed in the TgSwDI model with three autosomal dominant mutations (Swedish; Dutch; Iowa), but not in 4 or 5 month old J20 or 3xTg-AD mice. The accelerated deposition of Aβ in CAA in the TgSwDI model is in line with previous reports, and supports a critical role of the amino acid sequence and structure of Aβ in CAA formation [50, 70]. The Dutch and Iowa mutations are associated with extensive CAA but limited plaque pathology [74, 75].

In all transgenic mouse models investigated here, CAA contained npAβ and pSer8Aβ, indicating co-deposition of these variants. In a previous study, we showed co-deposition of phosphorylated and non-phosphorylated Aβ in the preclinical and clinical phase of AD [40]. However, about 30 to 40% of cases with CAA did not show pSer8Aβ [40], indicating a larger heterogeneity in the development of CAA in the human brain as compared to that of transgenic mouse models. As also demonstrated in this study, all investigated transgenic mouse models showed co-deposition of npAβ and pSer8Aβ in extracellular plaques apparently already at early phases of Aβ deposition in the respective model. The overall load pSer8Aβ in the transgenic mouse models appears to mainly depend on the extent of total Aβ generation determined by the individual mutations in APP or PS1. This is consistent with previous reports with other transgenic mouse models [18, 37]. However, as shown here and in previous studies [40, 42], only a subset of brains from human cases with Aβ plaque pathology, especially in the pathological preclinical phase of AD present with pSer8Aβ deposition. Together, these data on one hand show similarities in the deposition of modified and unmodified Aβ species, but on the other hand also demonstrate considerable differences in the composition of Aβ deposits during the pathogenesis of human AD and β-amyloidosis in transgenic mouse models.

## Conclusions

Previous studies showed that amyloid deposits of AD and DS brains are heterogeneous, and could contain post-translationally modified and truncated Aβ variants [16, 18–23, 29, 31, 35, 36, 39, 42, 54, 61, 76]. Some of these modified Aβ species are also observed in the brains of transgenic mice. These Aβ modifications are of particular interest because they could contribute to the deposition of Aβ by altering Aβ conformation, aggregation and the proteolytic degradation by neuropeptidases. Thus, the comparative analysis of the spatiotemporal deposition of pSer8Aβ and other modified or unmodified Aβ species, and the relative composition of characteristic Aβ deposits in form of extracellular plaques and cerebral amyloid angiopathy could be relevant for better understanding the onset and progression of AD and AD like pathology in DS, and to identify specific Aβ species for diagnosis and therapeutic targeting.

## Supplementary information

**Additional file 1: Supplementary Figure 1. Specificity of phosphorylation-state specific Aβ antibodies.** SDS-PAGE electrophoresis and immunoblotting of non-modified full length (Aβ1–40/42), truncated (Aβ3–40/42), phosphorylated (pSer8Aβ1–40/42, pSer26Aβ1–40, pSer8Aβ3–40/42, pSer26Aβ3–40/42), pyroglutaminated Aβ (pyroAβ3–40/42) or nitrated Aβ (NitroAβ1–42) variants with 1E4E11 (pSer8Aβ-specific) and 7H3D6 (npAβ-specific) antibodies. 1E4E11 specifically recognized phosphorylated Ser-8 full-length (1–40/42) and truncated pSer8Aβ3–40/42 variants, whereas 7H3D6 antibody demonstrated no reactivity against Aβ peptides phosphorylated at Ser-8 residue and specifically detects only full-length Aβ1–40/42 variants without N-terminal modifications. Generic 4G8-antibody (epitope 17–24) recognizes all examined Aβ variants.

**Additional file 2: Supplementary Figure 2. pSer8Aβ and npAβ deposition in parenchyma, leptomeningeal blood vessels and intraneuronally in different Tg mouse models.** Immunohistochemistry of 16 months old J20 (a-d), 14 month-old hAPP751 (e-h), 18 month-old PS/APP (i-l), and 27 month-old 3xTg-AD (m-p) mouse brain tissue demonstrates the presence of pSer8Aβ in leptomeningeal and parenchymal blood vessels in addition to extracellular amyloid plaques in J20, hAPP751 and PS/APP mouse brains, and extracellular and intraneuronal accumulation of npAβ and pSer8Aβ in the neocortex in 3xTg-AD mice. Boxes in images a, b, e, f, i, j, m and n (scale bar = 200 μm) are enlarged in c, d, g, h, k, l, o and p (scale bar = 50 μm).

## Data Availability

All data generated or analyzed during this study are included in this article and its supplementary files.

## Abbreviations

AD: Alzheimer’s disesae
Aβ: Amyloid-beta peptide
AC: Aged control
APP: Amyloid precursor protein
CAA: Cerebral amyloid angiopathy
DS: Down syndrome
FAD: Familial Alzheimer's disease
mAb: monoclonal antibody
MAPT: Microtubule-associated protein tau
npAβ: Non-phosphorylated Aβ
pSer8Aβ: Phosphorylated Serine-8 Aβ
PS: Presenilin
