# Supplementary figures and images for: Phosphorylated Aβ peptides in human Down syndrome brain and different Alzheimer’s-like mouse models
Source: Acta Neuropathol Commun. 2020 Jul 29;8:118. doi: 10.1186/s40478-020-00959-w (PMC7388542; doi:10.1186/s40478-020-00959-w)

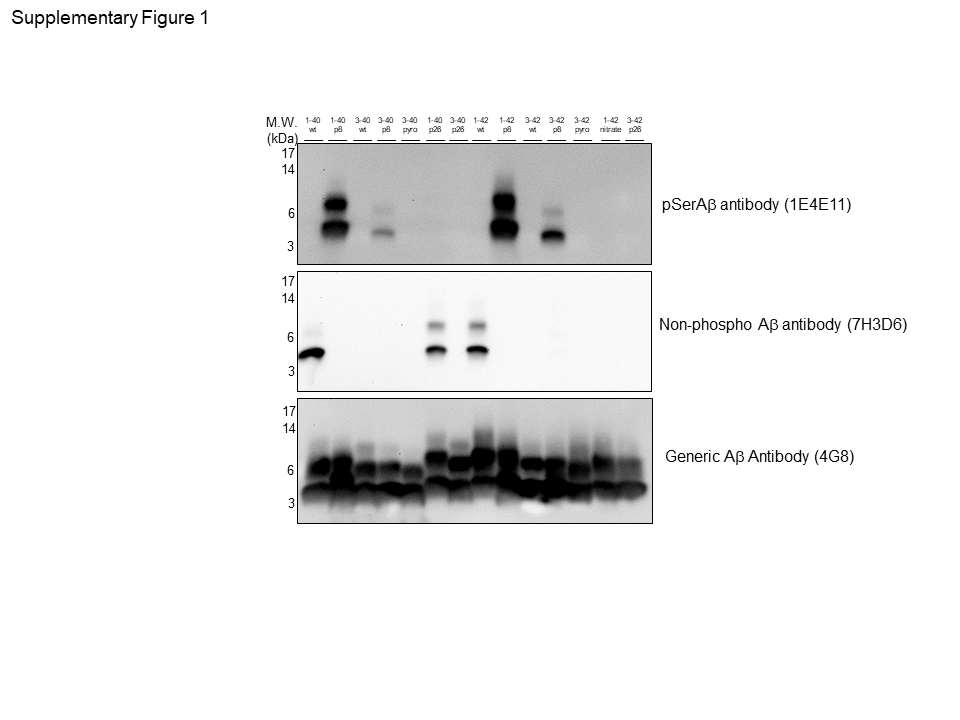

Supplement: Supplementary file 1 — Additional file 1: Supplementary Figure 1. Specificity of phosphorylation-state specific Aβ antibodies. SDS-PAGE electrophoresis and immunoblotting of non-modified full length (Aβ1–40/42), truncated (Aβ3–40/42), phosphorylated (pSer8Aβ1–40/42, pSer26Aβ1–40, pSer8Aβ3–40/42, pSer26Aβ3–40/42), pyroglutaminated Aβ (pyroAβ3–40/42) or nitrated Aβ (NitroAβ1–42) variants with 1E4E11 (pSer8Aβ-specific) and 7H3D6 (npAβ-specific) antibodies. 1E4E11 specifically recognized phosphorylated Ser-8 full-length (1–40/42) and truncated pSer8Aβ3–40/42 variants, whereas 7H3D6 antibody demonstrated no reactivity against Aβ peptides phosphorylated at Ser-8 residue and specifically detects only full-length Aβ1–40/42 variants without N-terminal modifications. Generic 4G8-antibody (epitope 17–24) recognizes all examined Aβ variants. [file 40478_2020_959_MOESM1_ESM.tif]
